# Data supporting the development of loading protocols for seismic qualification of BRBs considering global performance requirements

**DOI:** 10.1016/j.dib.2019.104803

**Published:** 2019-11-14

**Authors:** Mario Aguaguiña, Ying Zhou, Yun Zhou

**Affiliations:** a State Key Laboratory of Disaster Reduction in Civil Engineering, Tongji University, Shanghai 200092, China; b College of Civil Engineering, Guangzhou University, Guangzhou 510006, China

**Keywords:** Buckling-restrained braces, Loading protocol, Numerical simulation, OpenSees, Quasi-static cyclic test, Qualification testing

## Abstract

The data presented in this article constitute a supplementary material and provide support to the study *“Loading protocols for qualification testing of BRBs considering global performance requirements”* by Aguaguiña et al. [1]. Three types of datasets are given herein mainly in the form of tables. The first two correspond to databases built based on a compilation of past experimental testing on buckling-restrained braces (BRBs), conducted in different parts of the world. Two distinct objectives motivated such data compilation: (1) to determine a practical range for the parameter named as Yield Length Ratio (*YLR*), from full-scale and large-scale buckling-restrained braced frame (BRBF) specimens, that allowed for evaluation of the maximum deformation demands of BRBs [1]; and (2) to extract information on the properties of BRB specimens of different types, materials, and origin, for calibration of a numerical model that enabled the simulations of quasi-static cyclic tests of BRBs under five different code-prescribed loading histories (US, EU, CN, JP, and CA) and two proposed global loading protocols (GLP-1 and GLP-2) [1]. The last dataset type corresponds to the results of these numerical simulations, which were conducted using the OpenSees platform. The results show the maximum ductility demand (*μ*_max_), cumulative inelastic deformation, in terms of cumulative plastic ductility (*CPD*) and cumulative plastic strain (*CPS*), and cumulative hysteretic energy (*Ē*_*h*_) of BRBs as a result of the application of each loading sequence, and they are tabulated by BRB specimen.

**Table:** Specifications Table

Subject	*Civil and Structural Engineering*
Specific subject area	*Qualification testing of buckling-restrained braces (BRBs)*
Type of data	*Table, Figure*
How data was acquired	*Compilation of past experimental studies on BRBs and BRBFs; and numerical simulation of cyclic tests of BRBs using OpenSees*
Data format	*Tabulated, Analyzed*
Parameters for data collection	*Compilation of BRB tests: no pre-treatment of data.* *Simulation of BRB tests: force-deformation response data from analyses was used to compute some performance parameters presented here, i.e., maximum ductility demand (μ*_max_*); cumulative plastic ductility (CPD); cumulative plastic strain (CPS); and cumulative hysteretic energy (Ē*_*h*_*).*
Description of data collection	*Compilation of BRB tests:* *Data were retrieved from literature published between 2002 and 2018.* *Simulation of BRB tests:* *A model representing a BRB in a uniaxial test setup was established in OpenSees. The model was intended to capture the nonlinear behavior of BRBs under cyclic loading. The model was calibrated using the test data of* 35 BRB *specimens (material properties, dimensions, hysteresis loops, etc.). Then, with the calibrated model of each BRB specimen, static cyclic analyses were conducted by applying five different code-prescribed loading histories (i.e., US, EU, CN, JP, and CA). Additional analyses were conducted under two new loading sequences, namely global loading protocols (i.e., GLP-1 and GLP-2). Finally, key performance parameters were computed from force and deformation output data.*
Data source location	*Compilation of BRB tests: United States, Hungary, Turkey, China (incl. Taiwan), Japan, and Canada* *Simulation of BRB tests: Tongji University, Shanghai 200092, China*
Data accessibility	*All data (BRB test databases and simulation results) are provided within this article*
Related research article	Aguaguiña, M., Zhou, Y., Zhou, Y. Loading protocols for qualification testing of BRBs considering global performance requirements. *Engineering Structures* 2019; **189**:440–457. *Authors' names:* Mario Aguaguiña, Ying Zhou, Yun Zhou; *Title:* Loading protocols for qualification testing of BRBs considering global performance requirements; *Journal:* Engineering Structures; DOI: https://doi.org/10.1016/j.engstruct.2019.03.094

**Table:** 

**Value of the Data** • The compiled data gather several relevant experimental campaigns, conducted in different countries since 2000, on both individual BRBs and full-scale BRBFs. The test databases can be used a reference by researchers in this topic. • Based on retrieved data from past experiments of full-scale and large-scale BRBFs, the values that the design parameter named as Yield Length Ratio (*YLR*) takes in very detailed BRBF specimens were investigated. The 12 experimental campaigns on BRBFs cover different test setups (from one- to four-story frames), bracing configurations (diagonal, chevron), end-connections (bolted, pinned, welded), and testing methods (quasi-static, pseudo-dynamic, hybrid simulation). This permitted the definition of a practical range for the *YLR* in a realistic way with the purpose of determining the maximum deformation demands of BRBs, as further explained in Aguaguiña et al. [1]. • The database of past tests of individual BRBs was formed from 16 experimental studies conducted in the United States, Hungary, Turkey, China (incl. Taiwan), Japan, and Canada. From these experiments, 35 BRB specimens were selected with the intention of calibrating a model established in OpenSees for simulation of cyclic tests under different loading protocols. The specimen properties, loading histories used in the tests, and experimental force-deformation response was the main information retrieved from these studies. In addition, the data collected cover a reasonably wide range of variability in type, size/capacity, material strength, restrainer configuration, and origin of BRB devices that have been tested since an early stage of development of this technology until now. • Note that these two test databases are referred to but not shown in Aguaguiña et al. [1], so this data article constitutes an important supplementary material to the related research article [1]. • In Aguaguiña et al. [1], the 35 BRB specimens described above were classified by strength grade of the steel core material, resulting in three groups: low-yield point steel (G1); mild steel (G2); and high-strength steel (G3). So, analyses results were presented in a summarized form. The last two datasets provided in this data article contain an extended version of the results of the simulation of cyclic tests of BRBs under both code-prescribed loading histories and proposed global loading protocols; the results are listed by ‘specimen’ instead of by ‘specimen group’. Therefore, the data given herein complements the related research article [1] since full results can be consulted, and outcomes can be verified.

## 1 Data

The data in this brief constitute supplementary material and provides support to a research on testing protocols for seismic performance assessment and qualification of BRBs [1]. The data provided in this article are composed of six tables and one figure, which are briefly described as follows:

1. *Compilation of past experimental studies on BRBs and BRBFs:* Two datasets of this type are included in this article. Table 1, in Section 2.1, shows a dataset that lists 12 experimental tests of full-scale and large-scale BRBFs. The information in this table includes the following: source; specimen description (setup and bracing configuration); specimen size; testing method; end-connection type; yielding length; work point length; and yield length ratio. Table 2, in Section 2.2, shows a dataset that lists 35 BRB specimens selected from 16 experimental studies. The information in this table includes the following: source; specimen; BRB type; loading protocols used in testing; material specification and material properties; and specimen dimension and capacity.

**Table 1:** Values of the *YLR* in full-scale and large-scale BRBF specimens [[2], [3], [4], [5], [6], [7], [8], [9], [10], [11], [12], [13], [14], [15], [16], [17], [18], [19], [20], [21], [22], [23]].

Source	Specimen description	Specimen size	Testing method	BRB-to-gusset plate connection type	Yielding length, *L*_*y*_ (mm)	Work point length, *L*_*wp*_ (mm)	Yield length ratio, *YLR*
Mahin et al. [2], Field and Ko [3]	Two specimens: one-bay, one-story subassembly with chevron and diagonal bracing config.	Large-scale (*λ* = 0.7)	Quasi-static	Bolted	≈1850, ≈3430	4494, 6933	0.41, 0.50
Nishimoto et al. [4]	Four specimens. Inclined brace test: BRB fixed to a strong floor at the bottom and attached to the top free end of a pinned column	Full-scale	Quasi-static	Bolted	2747, 2907; 6018, 6178	6293; 9951	0.44, 0.46; 0.60, 0.62
Fahnestock et al. [[5], [6], [7]]	One-bay, four-story tall plus one-basement-story steel BRBF with inverted-V bracing configuration	Large-scale (*λ* = 0.6)	Hybrid pseudo-dynamic	Pinned	1651.0–1981.2	3570.8–3879.5	0.46–0.51
Tsai et al. [8], Chen et al. [9], Lin et al. [10], Tsai et al. [11], Tsai and Hsiao [12]	Two-phase test of a 3-story 3-bay buckling-restrained braced frame (BRBF) using concrete-filled steel tube columns (CFTs) 3-story 3-bay CFT/BRB frame	Full-scale	Pseudo-dynamic	Bolted	Phase-1: 2965, 2628, 3220; Phase-2: 2605, 2658, 2660	Phase-1: 5183, 5315, 5334; Phase-2: 5183, 5315, 5334	Phase-1: 0.57, 0.49, 0.60; Phase-2: 0.50, 0.50, 0.50
Weng et al. [13]	One-bay, two-story steel BRBF with diagonal bracing config.	Full-scale	3D sub-structural pseudo-dynamic	Welded, Bolted	6201, 5396	8802, 8947	0.70, 0.60
Berman and Bruneau [14]	One-bay, three-story steel BRBF with diagonal bracing config.	Small-scale (*λ* = 1/3)	Quasi-static	Bolted	994, 994, 994	2452, 2406, 2406	0.41, 0.41, 0.41
Lin et al. [15]	One-bay, three-story steel BRBF with V (story 2) and inverted-V (stories 1 and 3) bracing configuration	Full-scale	Hybrid test + Cyclic loading test	Welded	1677, 1752, 1752	4193, 4380, 4380	0.40, 0.40, 0.40
Takeuchi et al. [16,17]	Six specimens. Inclined brace test: BRB fixed to a reaction beam on top while attached to a movable lower jig at the bottom	Full-scale	Quasi-static cyclic loading test	Bolted	(3)1380; (3)1200	2700	0.51, 0.44
Sutcu et al. [18]	One-bay, one-story RC frame, representing a low-standard school building, retrofitted with BRBs	Near full-scale	Quasi-static cyclic loading test	Welded	1740	3525	0.50
Tsai et al. [19], Pan et al. [20]	One bay, one story reinforced concrete (RC) frame retrofitted with buckling-restrained braces	Full-scale	Quasi-static cyclic loading test	Welded	2705	4109	0.66
Dehghani and Tremblay [21]	Half span of a one-bay, one-story steel frame with V- bracing configuration	Full-scale	Pseudo-dynamic	Bolted	3000	6000	0.50
Tsai et al. [19], Wu et al. [22], Tsai et al. [23]	One-bay, two-story new reinforced concrete (RC) frame with BRBs, with diagonal bracing configuration 2-story 1-bay BRB-RC frame	Full-scale	Hybrid test + Cyclic loading test	Welded	3473, 3586	6025, 5980	0.58, 0.60

**Table 2:** Properties of selected BRB specimens for simulation of cyclic tests [21,[24], [25], [26], [27], [28], [29], [30], [31], [32], [33], [34], [35], [36], [37], [38], [39], [40], [41]].

Source	Specimen	BRB type	Loading protocol	Material specification	*F*_*ysc*_ (kN/mm^2^)	*ε*_*y*_ (%)	*A*_*sc*_ (mm^2^)	*L*_*y*_ (mm)	*P*_*ysc*_ (kN)	Δ_*by*_ (mm)
**United States**										
Merritt et al. [24]	1D	Typ.	AISC-SEAOC	ASTM A36	0.268	0.134	6451.6	3378.2	1725.8	4.52
	3D	Typ.	AISC-SEAOC	ASTM A36	0.303	0.152	10322.6	3327.4	3127.7	5.04
	5D	Typ.	AISC-SEAOC	ASTM A36	0.268	0.134	14919.3	3302.0	3990.9	4.42
Black et al. [25,26]	99–3	Typ.	SAC	JIS SM490A	0.419	0.209	5149.0	3450.0	2154.9	7.22
	00–12	Typ.	OSHPD	JIS SN400B	0.285	0.143	7125.0	3410.0	2033.5	4.87
**Europe: Hungary, Turkey**										
Dunai et al. [27]	EWC800A	Typ.	EN15129*	S235 JR	0.282	0.141	800.0	2000.0	225.6	2.82
	EWC800B	Typ.	EN15129*	S235 JR	0.282	0.141	800.0	2000.0	225.6	2.82
Ozcelik et al. [28]	BRB2	Typ.	AISC*	ASTM A36	0.293	0.147	2250.0	1703.0	659.3	2.49
	BRB4	Typ.	AISC*	ASTM A36	0.293	0.147	2250.0	1703.0	659.3	2.49
**China (including Taiwan)**										
Li et al. [29]; Sun et al. [30]	TJI-2	All-S	CN	Q195	0.226	0.110	7603.9	2600.0	1720.0	2.85
	TJII-1	Typ.	CN	BLY225	0.252	0.124	27797.6	8000.0	7005.0	9.88
Liu et al. [31]	1	Typ.	CN	Q235	0.284	0.139	6880.0	3000.0	1950.5	4.17
Huang et al. [32]	BRB-W-V1	All-S	CN	Q235	0.293	0.145	977.2	1074.0	286.5	1.56
Chou and Chen [33]	1	All-S	AISC	ASTM A572 Gr. 50	0.367	0.181	3300.0	2800.0	1211.1	5.06
Tsai et al. [34]	CR	Typ.	AISC*	JIS SM490B	0.380	0.190	2000.0	1000.0	760.0	1.90
	WES-R	Typ.	AISC*	JIS SM490B	0.334	0.167	4000.0	1700.0	1336.0	2.84
	WES-C	Typ.	AISC*	JIS SM490B	0.388	0.194	7525.0	2060.0	2919.7	4.00
	WES-J	Typ.	AISC*	JIS SM490B	0.400	0.190	26500.0	9500.0	10600.0	18.10
**Japan**										
Iwata [35]	Type 1	Typ.	JP-Iwata	JIS SN400B	0.263	0.131	2816.0	1800.0	739.5	2.36
Iwata and Murai [36]	P25S9	SMPs	JP-Iwata	JIS SN400B	0.295	0.148	1248.0	1176.0	368.2	1.73
	P23M7	SMPs	JP-Iwata	JIS SN400B	0.289	0.145	1664.0	1176.0	480.9	1.70
	P24L4	SMPs	JP-Iwata	JIS SN400B	0.278	0.139	1936.0	1176.0	538.2	1.63
	P23L5	SMPs	JP-Iwata	JIS SN400B	0.278	0.139	2288.0	1176.0	636.1	1.63
	P45M9	SMPs	JP-Iwata	JIS SN400B	0.289	0.145	2208.0	1176.0	638.1	1.70
Iwata et al. [37]	Type W	SMPs	JP-Iwata	JIS SN400B	0.277	0.139	1568.0	945.0	434.3	1.31
Midorikawa et al. [38]	L650S	SMPs	JP-Iwata	JIS SN400B	0.281	0.141	1584.0	2218.0	445.1	3.12
	L850S	SMPs	JP-Iwata	JIS SN400B	0.281	0.141	1584.0	2925.0	445.1	4.11
Takeuchi et al. [39,40]	CY110M15	Typ.	JP-Takeuchi	LY225	0.257	0.125	2080.0	1000.0	534.6	1.25
	CY138M15	Typ.	JP-Takeuchi	LY225	0.257	0.125	2080.0	1000.0	534.6	1.25
**Canada**										
Tremblay et al. [41]	C1-1	Typ.	NEHRP	G40.21–350WT	0.370	0.185	1587.5	2483.0	587.4	4.59
	C2-1	Typ.	NEHRP	G40.21–350WT	0.370	0.185	1587.5	1001.0	587.4	1.85
Dehghani and Tremblay [21]	S9	All-S	AISC*	G40.21–350WT	0.385	0.183	2840.3	3000.0	1093.5	5.50
	S10	All-S	AISC*	G40.21–350WT	0.385	0.183	2827.2	3000.0	1088.5	5.50
	S11	All-S	AISC*	G40.21–350WT	0.385	0.183	2863.2	3000.0	1102.3	5.50
	S12	All-S	AISC*	G40.21–350WT	0.385	0.183	2825.8	3000.0	1088.0	5.50

Typ. = typical BRB, whose restraining system is composed by a steel tube filled with mortar/concrete surrounding the steel core; All-S = all-steel BRB or “dry” BRB, whose restraining mechanism is composed by steel plates or sections, adapted to the shape of the steel core, typically assembled by bolting; SMPs = BRBs, first proposed by Iwata and Murai [36], whose restraining system is composed by a pair of prefabricated mortar-filled steel channels (referred to as steel mortar planks, SMPs) assembled by welding.

**Fig. 1:**
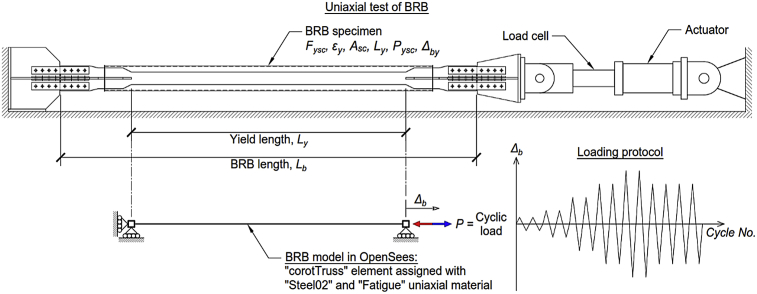
Nonlinear model of BRB for cyclic analyses under different loading protocols. From Aguaguiña et al. [1].

AISC* = customized AISC loading protocol; EN15129* = customized EN15129 loading protocol.
2. *Numerical simulation of cyclic tests of BRBs:* These data are given in the form of two tables. Table 5, in Section 2.3.1, presents the results from numerical simulation of cyclic tests of BRB under five code-prescribed loading protocols, namely US, EU, CN, JP, and CA. Table 6, in Section 2.3.2, shows the results obtained from computational simulation of cyclic tests of BRBs under two proposed global loading protocols, namely GLP-1 and GLP-2. Both tables list the same results, as follows: maximum ductility demand, *μ*_max_; cumulative plastic ductility, *CPD*; cumulative plastic strain, *CPS*; and cumulative hysteretic energy, *Ē*_*h*_*.*
3. *Auxiliary material:* Fig. 1, Table 3, and Table 4 are relevant contents in Section 2.3 that help to fully understand the datasets corresponding to analyses results described in the previous point.

**Table 3:** US, EU, CN, JP, and CA loading protocols for BRB testing expressed in terms of *ε*_*b,sc*_. From Aguaguiña et al. [1].

Loading protocol		Number of cycles per deformation amplitude, *ε*_*b,sc*_						Total num. of cycles
		1	2	3	4	5	6	
US	*YLR* = 0.50	2@*ε*_*y*_	2@1.0%	2@2.0%	2@3.0%	2@4.0%	–	10 cycles
	YLR = 0.60	2@*ε*_*y*_	2@0.83%	2@1.65%	2@2.48%	2@3.3%	–	
EU	*YLR* = 0.50	5@0.5%	5@1.0%	10@2.0%	–	–	–	20 cycles
	YLR = 0.60	5@0.41%	5@0.83%	10@1.65%	–	–	–	
CN	*YLR* = 0.50	3@*ε*_*y*_	3@0.67%	3@1.0%	3@1.33%	3@2.0%	3@2.4%	18 cycles
	YLR = 0.60	3@*ε*_*y*_	3@0.55%	3@0.83%	3@1.1%	3@1.65%	3@1.98%	
JP	–	3@*ε*_*y*_	3@0.5%	3@1.0%	3@2.0%	3@3.0%	–	15 cycles
CA	–	8@*ε*_*y*_	6@1.5*ε*_*y*_	4@2.5*ε*_*y*_	3@3.5*ε*_*y*_	2@4.5*ε*_*y*_	2@5.5*ε*_*y*_	25 cycles

*ε*_*b,sc*_ = axial strain of steel core of BRB = Δ_*b*_/*L*_*y*_.

*ε*_*y*_ = core strain, *ε*_*b,sc*_, at first yield of test specimen.

**Table 4:** Definition of proposed global loading protocols for seismic qualification of BRBs. From Aguaguiña et al. [1].

Loading protocol		Number of cycles per deformation amplitude, *ε*_*b,sc*_						Total num. of cycles
		1	2	3	4	5	6	
			0.25*a*_max_	0.45*a*_max_	0.6*a*_max_	0.75*a*_max_	*a*_max_	
GLP-1	*YLR* = 0.50	3@*ε*_*y*_	3@1.0%	2@1.8%	2@2.4%	2@3.0%	2@4.0%	14 cycles
GLP-2	*YLR* = 0.60	3@*ε*_*y*_	3@0.8%	2@1.5%	2@2.0%	2@2.5%	2@3.3%	14 cycles

*ε*_*b,sc*_ = axial strain of steel core of BRB = Δ_*b*_/*L*_*y*_.

*ε*_*y*_ = core strain, *ε*_*b,sc*_, at first yield of test specimen.

**Table 5:** Simulation results: maximum and cumulative demands imposed by US, EU, CN, JP, and CA loading protocols. Listed by specimen.

Source	Specimen	*YLR*	US loading protocol				EU loading protocol				CN loading protocol			
			*μ*_max_	*CPD*	*CPS* (%)	*Ē*_*h*_	*μ*_max_	*CPD*	*CPS* (%)	*Ē*_*h*_	*μ*_max_	*CPD*	*CPS* (%)	*Ē*_*h*_
**United States**														
Merritt et al. [24]	1D	0.50	29.9	566	75.7	811.0	15.0	742	99.3	875.4	17.9	604	80.8	719.4
		0.60	24.7	461	61.7	615.1	12.3	599	80.1	663.8	14.8	488	65.2	546.0
	3D	0.50	26.4	496	75.2	629.2	13.2	646	97.9	711.3	15.8	526	79.7	583.5
		0.60	21.8	404	61.2	485.8	10.9	519	78.6	546.4	13.1	424	64.2	448.9
	5D	0.50	29.9	566	75.7	811.0	15.0	742	99.3	875.4	17.9	604	80.8	719.4
		0.60	24.7	461	61.7	615.1	12.3	599	80.1	663.8	14.8	488	65.2	546.0
Black et al. [25,26]	99–3	0.50	19.1	350	73.3	377.3	9.6	446	93.3	427.7	11.5	364	76.2	351.6
		0.60	15.8	283	59.3	292.7	7.9	354	74.0	327.7	9.5	290	60.7	270.0
	00–12	0.50	28.0	529	75.4	718.6	14.0	691	98.6	794.8	16.8	562	80.2	652.6
		0.60	23.1	431	61.4	550.0	11.6	556	79.3	606.8	13.9	453	64.7	498.8
**Europe**														
Dunai et al. [27]	EWC800A	0.50	28.4	535	75.5	750.9	14.2	700	98.7	810.6	17.0	570	80.3	666.3
		0.60	23.4	436	61.5	569.8	11.7	564	79.5	614.5	14.0	460	64.8	505.7
	EWC800B	0.50	28.4	535	75.5	737.8	14.2	700	98.7	795.3	17.0	570	80.3	653.8
		0.60	23.4	436	61.5	559.5	11.7	564	79.5	603.1	14.0	460	64.8	496.3
Ozcelik et al. [28]	BRB2	0.50	27.3	514	75.3	613.3	13.7	671	98.3	707.3	16.4	546	80.0	579.7
		0.60	22.5	419	61.3	477.1	11.3	539	79.0	546.9	13.5	440	64.5	448.8
	BRB4	0.50	27.3	514	75.3	613.3	13.7	671	98.3	707.3	16.4	546	80.0	579.7
		0.60	22.5	419	61.3	477.1	11.3	539	79.0	546.9	13.5	440	64.5	448.8
**China (including Taiwan)**														
Li et al. [29]; Sun et al. [30]	TJI-2	0.50	36.4	697	76.5	1153.4	18.2	922	101.2	1271.3	21.9	749	82.2	1041.8
		0.60	30.1	569	62.5	878.2	15.0	746	82.0	973.4	18.0	607	66.7	798.4
	TJII-1	0.50	32.4	616	76.0	931.1	16.2	810	100.1	1071.0	19.4	659	81.4	875.2
		0.60	26.7	502	62.0	719.6	13.4	655	80.9	831.9	16.0	533	65.8	680.5
Liu et al. [31]	1	0.50	28.8	544	75.6	943.7	14.4	712	98.9	1030.3	17.3	579	80.5	844.8
		0.60	23.7	443	61.6	716.3	11.9	573	79.6	785.2	14.2	467	64.9	644.3
Huang et al. [32]	BRB-W-V1	0.50	27.6	519	75.4	604.0	13.8	678	98.4	691.9	16.5	552	80.1	567.2
		0.60	22.7	423	61.4	468.8	11.4	545	79.1	534.1	13.6	445	64.6	438.4
Chou and Chen [33]	1	0.50	22.1	411	74.2	477.9	11.1	528	95.5	533.0	13.3	431	78.0	438.0
		0.60	18.3	333	60.2	367.9	9.1	422	76.3	407.3	11.0	345	62.4	335.3
Tsai et al. [34]	CR	0.50	21.1	389	73.9	446.2	10.5	499	94.8	496.9	12.6	407	77.4	408.5
		0.60	17.4	315	59.9	343.5	8.7	398	75.6	379.3	10.4	326	61.9	312.4
	WES-R	0.50	24.0	447	74.7	501.7	12.0	579	96.6	572.5	14.4	472	78.8	469.8
		0.60	19.8	363	60.7	389.3	9.9	463	77.4	440.8	11.9	379	63.2	362.3
	WES-C	0.50	20.6	380	73.8	411.8	10.3	487	94.5	467.2	12.4	398	77.2	383.8
		0.60	17.0	308	59.8	319.3	8.5	388	75.2	358.5	10.2	318	61.6	295.1
	WES-J	0.50	21.0	388	73.9	443.0	10.5	498	94.8	509.0	12.6	406	77.4	417.9
		0.60	17.3	315	59.9	344.9	8.7	396	75.5	391.7	10.4	325	61.8	322.3
**Japan**														
Iwata [35]	Type 1	0.50	30.5	577	75.8	724.5	15.2	758	99.5	807.1	18.3	616	80.9	662.3
		0.60	25.1	471	61.8	556.2	12.6	611	80.2	618.2	15.1	498	65.4	507.8
Iwata and Murai [36]	P25S9	0.50	27.1	510	75.3	571.7	13.6	666	98.2	652.0	16.3	542	80.0	534.6
		0.60	22.4	415	61.3	443.1	11.2	535	79.0	503.2	13.4	437	64.4	413.2
	P23M7	0.50	27.7	522	75.4	622.1	13.8	681	98.4	689.9	16.6	555	80.1	566.5
		0.60	22.8	425	61.4	477.2	11.4	548	79.2	527.5	13.7	447	64.6	433.6
	P24L4	0.50	28.8	544	75.6	696.1	14.4	711	98.9	757.2	17.3	579	80.5	622.3
		0.60	23.7	443	61.6	530.0	11.9	573	79.6	575.7	14.2	467	64.9	473.6
	P23L5	0.50	28.8	544	75.6	671.3	14.4	711	98.9	748.8	17.3	579	80.5	614.5
		0.60	23.7	443	61.6	515.8	11.9	573	79.6	573.6	14.2	467	64.9	471.3
	P45M9	0.50	27.7	522	75.4	622.1	13.8	681	98.4	689.9	16.6	555	80.1	566.5
		0.60	22.8	425	61.4	477.2	11.4	548	79.2	527.5	13.7	447	64.6	433.6
Iwata et al. [37]	Type W	0.50	28.9	546	75.6	827.0	14.4	714	98.9	881.4	17.3	581	80.5	725.0
		0.60	23.8	445	61.6	624.4	11.9	575	79.7	665.3	14.3	469	65.0	547.7
Midorikawa et al. [38]	L650S	0.50	28.5	537	75.5	697.5	14.2	703	98.8	782.2	17.1	572	80.4	641.5
		0.60	23.5	438	61.5	536.5	11.7	566	79.5	600.5	14.1	461	64.8	493.0
	L850S	0.50	28.5	537	75.5	695.8	14.2	703	98.8	783.0	17.1	572	80.4	642.1
		0.60	23.5	438	61.5	535.9	11.7	566	79.5	601.4	14.1	461	64.8	493.8
Takeuchi et al. [39,40]	CY110M15	0.50	31.9	606	76.0	845.2	16.0	797	100.0	929.9	19.1	648	81.3	763.2
		0.60	26.3	494	62.0	645.3	13.2	644	80.7	709.7	15.8	524	65.7	583.1
	CY138M15	0.50	31.9	606	76.0	845.2	16.0	797	100.0	929.9	19.1	648	81.3	763.2
		0.60	26.3	494	62.0	645.3	13.2	644	80.7	709.7	15.8	524	65.7	583.1
**Canada**														
Tremblay et al. [41]	C1-1	0.50	21.6	400	74.1	542.9	10.8	515	95.2	592.6	13.0	420	77.7	487.6
		0.60	17.8	325	60.1	414.3	8.9	411	76.0	449.1	10.7	336	62.2	370.2
	C2-1	0.50	21.6	400	74.1	517.0	10.8	515	95.2	573.8	13.0	420	77.7	471.8
		0.60	17.8	325	60.1	397.1	8.9	411	76.0	437.2	10.7	336	62.2	360.1
Dehghani and Tremblay [21]	S9	0.50	21.8	404	74.1	510.8	10.9	520	95.3	583.3	13.1	424	77.8	479.7
		0.60	18.0	328	60.1	397.5	9.0	415	76.1	446.9	10.8	340	62.3	368.5
	S10	0.50	21.8	404	74.1	510.8	10.9	520	95.3	583.3	13.1	424	77.8	479.7
		0.60	18.0	328	60.1	397.5	9.0	415	76.1	446.9	10.8	340	62.3	368.5
	S11	0.50	21.8	404	74.1	500.3	10.9	520	95.3	568.4	13.1	424	77.8	467.5
		0.60	18.0	328	60.1	388.6	9.0	415	76.1	435.0	10.8	340	62.3	358.7
	S12	0.50	21.8	404	74.1	510.8	10.9	520	95.3	583.3	13.1	424	77.8	479.7
		0.60	18.0	328	60.1	397.5	9.0	415	76.1	446.9	10.8	340	62.3	368.5

Source	Specimen	*YLR*	JP loading protocol				CA loading protocol			
			*μ*_max_	*CPD*	*CPS* (%)	*Ē*_*h*_	*μ*_max_	*CPD*	*CPS* (%)	*Ē*_*h*_
**United States**										
Merritt et al. [24]	1D	–	22.4	535	71.6	685.3	5.5	130	17.4	112.1
	3D	–	19.8	467	70.7	545.5	5.5	130	19.7	115.2
	5D	–	22.4	535	71.6	685.3	5.5	130	17.4	112.1
Black et al. [25,26]	99–3	–	14.3	325	68.0	327.9	5.5	130	27.2	110.7
	00–12	–	21.0	499	71.2	615.4	5.5	130	18.6	114.7
**Europe**										
Dunai et al. [27]	EWC800A	–	21.3	505	71.2	634.6	5.5	130	18.3	112.0
	EWC800B	–	21.3	505	71.2	623.2	5.5	130	18.3	110.9
Ozcelik et al. [28]	BRB2	–	20.5	484	71.0	537.9	5.5	130	19.0	112.9
	BRB4	–	20.5	484	71.0	537.9	5.5	130	19.0	112.9
**China (including Taiwan)**										
Li et al. [29]; Sun et al. [30]	TJI-2	–	27.3	662	72.7	985.3	5.5	130	14.3	92.2
	TJII-1	–	24.3	583	72.1	814.6	5.5	130	16.1	81.5
Liu et al. [31]	1	–	21.6	513	71.3	801.6	5.5	130	18.1	86.4
Huang et al. [32]	BRB-W-V1	–	20.7	489	71.0	527.9	5.5	130	18.9	109.1
Chou and Chen [33]	1	–	16.6	383	69.3	411.5	5.5	130	23.5	110.2
Tsai et al. [34]	CR	–	15.8	363	68.9	383.9	5.5	130	24.7	110.1
	WES-R	–	18.0	419	70.0	437.6	5.5	130	21.7	109.0
	WES-C	–	15.5	354	68.7	358.0	5.5	130	25.2	109.0
	WES-J	–	15.8	362	68.9	387.6	5.5	130	24.8	115.6
**Japan**										
Iwata [35]	Type 1	–	22.8	546	71.7	623.3	5.5	130	17.1	106.5
Iwata and Murai [36]	P25S9	–	20.3	481	70.9	498.7	5.5	130	19.2	106.8
	P23M7	–	20.8	492	71.1	534.0	5.5	130	18.8	104.3
	P24L4	–	21.6	513	71.3	591.1	5.5	130	18.1	105.3
	P23L5	–	21.6	513	71.3	578.0	5.5	130	18.1	107.4
	P45M9	–	20.8	492	71.1	534.0	5.5	130	18.8	104.3
Iwata et al. [37]	Type W	–	21.7	515	71.4	693.8	5.5	130	18.0	115.2
Midorikawa et al. [38]	L650S	–	21.4	507	71.3	602.2	5.5	130	18.3	100.2
	L850S	–	21.4	507	71.3	601.8	5.5	130	18.3	106.8
Takeuchi et al. [39,40]	CY110M15	–	23.9	574	72.0	721.8	5.5	130	16.3	112.2
	CY138M15	–	23.9	574	72.0	721.8	5.5	130	16.3	112.2
**Canada**										
Tremblay et al. [41]	C1-1	–	16.2	374	69.1	461.3	5.5	130	24.1	120.5
	C2-1	–	16.2	374	69.1	443.5	5.5	130	24.1	119.4
Dehghani and Tremblay [21]	S9	–	16.4	377	69.2	445.3	5.5	130	23.8	162.7
	S10	–	16.4	377	69.2	445.3	5.5	130	23.8	162.7
	S11	–	16.4	377	69.2	435.1	5.5	130	23.8	158.5
	S12	–	16.4	377	69.2	445.3	5.5	130	23.8	162.7

**Table 6:** BRB Simulation results: maximum and cumulative demands imposed by GLP-1 and GLP-2 loading protocols. Listed by specimen.

Source	Specimen	Global loading protocol, GLP-1, YLR = 0.50				Global loading protocol, GLP-2, YLR = 0.60			
		*μ*_max_	*CPD*	*CPS* (%)	*Ē*_*h*_	*μ*_max_	*CPD*	*CPS* (%)	*Ē*_*h*_
**United States**									
Merritt et al. [24]	1D	29.9	716	95.7	1001.1	24.7	584	78.1	762.7
	3D	26.4	627	94.9	781.2	21.8	510	77.3	605.3
	5D	29.9	716	95.7	1001.1	24.7	584	78.1	762.7
Black et al. [25,26]	99–3	19.1	442	92.4	468.6	15.8	357	74.8	364.5
	00–12	28.0	668	95.3	889.8	23.1	545	77.7	683.9
**Europe**									
Dunai et al. [27]	EWC800A	28.4	677	95.4	926.8	23.4	552	77.8	706.4
	EWC800B	28.4	677	95.4	910.3	23.4	552	77.8	693.5
Ozcelik et al. [28]	BRB2	27.3	650	95.2	763.3	22.5	529	77.6	595.6
	BRB4	27.3	650	95.2	763.3	22.5	529	77.6	595.6
**China (including Taiwan)**									
Li et al. [29]; Sun et al. [30]	TJI-2	36.4	881	96.8	1427.9	30.1	721	79.2	1093.0
	TJII-1	32.4	778	96.2	1158.3	26.7	636	78.6	899.5
Liu et al. [31]	1	28.8	687	95.5	1167.0	23.7	560	77.9	890.4
Huang et al. [32]	BRB-W-V1	27.6	656	95.2	750.9	22.7	535	77.6	584.7
Chou and Chen [33]	1	22.1	518	93.6	592.2	18.3	421	76.0	457.4
Tsai et al. [34]	CR	21.1	491	93.2	552.9	17.4	398	75.6	427.0
	WES-R	24.0	564	94.3	623.4	19.8	459	76.7	485.2
	WES-C	20.6	480	93.1	511.4	17.0	389	75.5	397.7
	WES-J	21.0	489	93.2	551.2	17.3	397	75.6	430.3
**Japan**									
Iwata [35]	Type 1	30.5	730	95.8	897.5	25.1	596	78.2	691.8
Iwata and Murai [36]	P25S9	27.1	645	95.1	710.1	22.4	525	77.5	552.2
	P23M7	27.7	659	95.2	770.1	22.8	537	77.6	593.0
	P24L4	28.8	687	95.5	859.7	23.7	560	77.9	657.4
	P23L5	28.8	687	95.5	831.7	23.7	560	77.9	541.5
	P45M9	27.7	659	95.2	770.1	22.8	537	77.6	593.0
Iwata et al. [37]	Type W	28.9	690	95.5	1019.4	23.8	562	77.9	773.3
Midorikawa et al. [38]	L650S	28.5	679	95.4	864.9	23.5	554	77.8	667.8
	L850S	28.5	679	95.4	863.2	23.5	554	77.8	667.4
Takeuchi et al. [39,40]	CY110M15	31.9	766	96.1	1045.7	26.3	626	78.5	801.8
	CY138M15	31.9	766	96.1	1045.7	26.3	626	78.5	801.8
**Canada**									
Tremblay et al. [41]	C1-1	21.6	505	93.5	671.4	17.8	410	75.9	514.4
	C2-1	21.6	505	93.5	640.7	17.8	410	75.9	493.7
Dehghani and Tremblay [21]	S9	21.8	510	93.5	635.3	18.0	414	75.9	495.5
	S10	21.8	510	93.5	635.3	18.0	414	75.9	495.5
	S11	21.8	510	93.5	621.7	18.0	414	75.9	484.1
	S12	21.8	510	93.5	635.3	18.0	414	75.9	495.5

## 2 Experimental design, materials, and methods

The following subsections provide a complete description of the methods used for acquisition of the data shared in this article.

### 2.1 Database of past experimental tests of full-scale and large-scale BRBFs

As pointed out in Aguaguiña et al. [1], the values for the parameter *YLR* typically range from 0.50 to 0.70 in actual buckling-restrained braced frames (BRBFs), depending on the type of end-connections used (i.e., bolted, pinned, welded) and bracing configuration employed (diagonal or chevron). Since the range over which *YLR* is evaluated directly influences the relative deformation of the BRB, according to Equations (1)–(3) in Aguaguiña et al. [1], it is important to define it in a more realistic way. To this end, the study relied on publicly available data from past experimental tests on BRBFs conducted at a full-scale and large-scale. A total of 12 relevant experimental campaigns, published between 2004 and 2018 [[2], [3], [4], [5], [6], [7], [8], [9], [10], [11], [12], [13], [14], [15], [16], [17], [18], [19], [20], [21], [22], [23]], were compiled to investigate the values that the *YLR* take in very detailed BRBF specimens. Table 1 summarizes the information regarding the 12 experimental studies.

### 2.2 Database of past cyclic tests of individual BRBs

In Aguaguiña et al. [1], a total of 35 BRB specimens were selected from 16 experimental investigations and tests published between 2002 and 2018 [21,[24], [25], [26], [27], [28], [29], [30], [31], [32], [33], [34], [35], [36], [37], [38], [39], [40], [41]]. These tests were conducted in the United States (2), Hungary (1), Turkey (1), China (3), Taiwan (2), Japan (5), and Canada (2). The tests database includes typical BRBs (20), all-steel BRBs (7), and BRBs with steel mortar planks (8). Further, the dissipative core of the BRBs was made either of low-yield-point steel (4), mild steel (19) or high-strength steel (12) material. Table 2 summarizes the information regarding the 16 experimental studies and the properties of the 35 selected specimens, and it constitutes the analysis matrix of the referred study [1]. The information listed in Table 2 includes: source of data; specimen denomination; loading protocol used for tests; BRB core material specification; steel core material properties (*F*_*ysc*_, *ε*_*y*_); steel core geometry (*A*_*sc*_, *L*_*y*_); and BRB yield force and yield deformation (*P*_*ysc*_, Δ_*by*_).

### 2.3 Numerical simulation of cyclic tests of BRBs under different loading protocols

The *Open System for Earthquake Engineering Simulation* (OpenSees) platform [42] was employed for both modeling and numerical simulations of cyclic tests of BRBs. The model was built considering the typical setup for uniaxial cyclic tests of BRBs. It consisted of a two-node truss element with a length equal to that of the yielding segment of the BRB specimen, *L*_*y*_. That is, following the assumption that most of elastic and inelastic deformations take place within the yielding segment of the BRB, small elastic deformations in the transition and connection zones were totally neglected. The BRB model is illustrated in Fig. 1. For further details of the modeling and calibration procedure, the reader is referred to Aguaguiña et al. [1].

As described in Aguaguiña et al. [1], five different code-prescribed loading protocols were evaluated in this study: United States (US); Europe (EU); China (CN); Japan (JP); and Canada (CA). To quantify and compare the demands imposed to BRBs by the five different loading histories, it was convenient to specify the deformation amplitudes, Δ_*b*_, in terms of a common quantity. In this work, all the loading protocols were set in terms of the steel core strain, *ε*_*b,sc*_, which only depends on the geometric properties of the BRB specimen (i.e., *L*_*y*_). Thus, the US, EU, and CN loading sequences, whose deformation amplitudes were indexed as a function of the BRB design deformation, Δ_*bd*_, needed to be converted so that the deformation levels were also presented in terms of *ε*_*b,sc*_, as the JP and CA loading protocols. Here, two important assumptions were made for estimation of the maximum design demand of BRBs: (1) the design inter-story drift ratio equals the drift limitation in the US, EU, and CN codes; and (2) the design deformation demand, Δ_*bd*_, is evaluated considering two values of the parameter *YLR*: 0.50 and 0.60. Table 3 presents the US, EU, CN, JP, and CA loading histories, in terms of *ε*_*b,sc*_, used for simulations. Additional details on the definition of the loading histories for analyses can be found in Aguaguiña et al. [1].

In Aguaguiña et al. [1], two new loading sequences, named as GLP-1 and GLP-2, were proposed as global loading protocols for qualification testing of BRBs. These loading histories were derived based on (1) the review of the background and definition of loading protocols for cyclic tests of BRB prescribed in different codes, and (2) the results from numerical simulation of cyclic tests of BRBs under five different loading regimes (i.e., US, EU, CN, JP, and CA). GLP-1 constitutes a loading protocol for an upper level of demand (*YLR* = 0.50) whereas the loading history GLP-2 represents a lower level of demand (*YLR* = 0.60). Both loading histories were configured so that a series of conditions and criteria were satisfied (refer to Aguaguiña et al. [1]). GLP-1 and GLP-2 loading protocols are defined in Table 4.

Regarding to analyses results, the force and deformation responses resulting from the application of different loading sequences constituted the main output data from numerical simulations. From the force and deformation data corresponding to each BRB specimen and loading protocol, other performance parameters were computed, including the maximum ductility demand, *μ*_max_, cumulative inelastic deformation, in terms of cumulative plastic ductility, *CPD*, and cumulative plastic strain, *CPS*, and cumulative dissipated energy, *Ē*_*h*_. The formulae for computation of *μ*_max_, *CPD*, *CPS*, and *Ē*_*h*_ are given in Equations (4) through (7) in Aguaguiña et al. [1]. The post-processing of analyses results was done by using the MATLAB software. Taking into account that the test database is composed of 35 BRBs specimens, and that this study considered five code-prescribed loading protocols (i.e., US, EU, CN, JP, and CA) with two analysis cases for US, EU, and CN, a total of 280 static cyclic loading analyses were performed in OpenSees. On the other hand, the number of static cyclic loading analyses under the two proposed global loading protocols (i.e., GLP-1 and GLP-2) accounted for 70. These results are presented in this article as two datasets. Table 5 presents the results of numerical simulation of cyclic tests of BRBs with code-prescribed loading histories, whereas Table 6 presents the results corresponding to the analyses with the proposed global loading protocols.

#### 2.3.1 Results of simulation of cyclic tests of BRBs under code-prescribed loading histories

Table 5 presents the results from numerical simulations of cyclic tests of BRBs under the US, EU, CN, JP, and CA loading histories. The maximum steel core ductility as well as the cumulative imposed demands are reported for each of the 35 BRB specimens considered in Aguaguiña et al. [1].

#### 2.3.2 Results of simulation of cyclic tests of BRBs under proposed global loading protocols

Table 6 presents the results from numerical simulations of cyclic tests of BRBs under the two proposed global loading protocols, GLP-1 and GLP-2. The maximum steel core ductility as well as the cumulative imposed demands are reported for each of the 35 BRB specimens considered in Aguaguiña et al. [1].
